# Unveiling the true burden of endodontic adverse events in academic settings: A five-year multivariate risk analysis

**DOI:** 10.21142/2523-2754-1403-2026-301

**Published:** 2026-07-10

**Authors:** Lilian Cortines-Acosta, Claudia Negrete-Romero, Javier Alvear-Pérez, Jaime Plazas-Román, Antonio Díaz-Caballero

**Affiliations:** 1 Faculty of Dentistry, University of Cartagena. Cartagena, Colombia. lcortinesa@gmail.com, , cnegrete.od.06@hotmail.com , jalvearp@unicartagena.edu.co, , adiazc1@unicartagena.edu.co Faculty of Dentistry University of Cartagena Cartagena Colombia lcortinesa@gmail.com cnegrete.od.06@hotmail.com jalvearp@unicartagena.edu.co adiazc1@unicartagena.edu.co

**Keywords:** adverse events, endodontics, risk factors, multivariate analysis, patient safety, efectos adversos, endodoncia, factores de riesgo, análisis multivariante, seguridad del paciente

## Abstract

**Background:**

Adverse events in endodontics remain an underexplored aspect of patient safety, with limited research employing robust multivariate methodologies to identify contributing risk factors.

**Material and Methods:**

Retrospective cohort study conducted in the postgraduate endodontics clinics at the University of Cartagena. All included records required a minimum six-month post-treatment follow-up, tracking the same patients from treatment to outcome, which supports the cohort design classification. From a total of 2,852 clinical records, a representative sample of 384 was selected. Variables analyzed included patient demographics, treatment type, case complexity, and provider training level. Statistical analyses comprised descriptive statistics, chi-square testing, stepwise multivariate logistic regression, Kaplan-Meier survival curves, Cox regression, and model validation through bootstrap resampling and cross-validation.

**Results:**

Among the 384 records reviewed, the incidence of adverse events was 15.1% (95%CI: 11.8-19.0%; n=58). The most frequent event was instrument fracture (62.1%, n=36), followed by over-obturation (20.7%, n = 12) and perforation (17.2%, n = 10). Multivariate analysis identified four independent risk factors: third-semester versus first-semester students (OR = 2.84; 95%CI: 1.52-5.31; p = 0.001), patient age 30-39 versus <30 years (OR = 2.12; 95%CI: 1.18-3.81; p = 0.012), retreatment versus initial treatment (OR = 3.67; 95%CI: 1.94-6.95; p < 0.001), and molar versus anterior teeth (OR = 2.45; 95%CI: 1.28-4.69; p = 0.007). The predictive model demonstrated strong discriminatory power (C-statistic = 0.782; 95%CI: 0.731-0.833) and good calibration (Hosmer-Lemeshow p = 0.622).

**Conclusions:**

The true incidence of adverse events in academic endodontics is notably higher than previously reported. The identified risk factors support the implementation of targeted preventive strategies and inform educational supervision and patient safety protocols.

## RESUMEN

**Antecedentes:**

Los eventos adversos en endodoncia constituyen un aspecto poco explorado de la seguridad del paciente, con investigación limitada que emplee metodologías multivariadas robustas para identificar los factores de riesgo contribuyentes.

**Material y métodos:**

Estudio de cohorte retrospectivo realizado en las clínicas de posgrado de endodoncia de la Universidad de Cartagena. De un total de 2852 registros clínicos, se seleccionó una muestra representativa de 384. Las variables analizadas incluyeron demografía del paciente, tipo de tratamiento, complejidad del caso y nivel de formación del operador. Los análisis estadísticos comprendieron estadística descriptiva, prueba de chi-cuadrado, regresión logística multivariada por pasos, curvas de supervivencia de Kaplan-Meier, regresión de Cox y validación del modelo mediante remuestreo bootstrap y validación cruzada.

**Resultados:**

Entre los 384 registros revisados, la incidencia de eventos adversos fue del 15,1% (IC95%: 11,8-19,0%; n = 58). El evento más frecuente fue la fractura de instrumentos (62,1%, n = 36), seguido de sobreobturación (20,7%, n = 12) y perforación (17,2%, n = 10). El análisis multivariado identificó cuatro factores de riesgo independientes: estudiantes de tercer semestre versus primer semestre (OR = 2,84; IC95%: 1,52-5,31; p = 0,001), edad del paciente 30-39 versus <30 años (OR = 2,12; IC95%: 1,18-3,81; p = 0,012), retratamiento versus tratamiento inicial (OR = 3,67; IC95%: 1,94-6,95; p < 0,001), y dientes molares versus anteriores (OR = 2,45; IC95%: 1,28-4,69; p = 0,007). El modelo predictivo demostró un fuerte poder discriminatorio (estadístico C = 0,782; IC95%: 0,731-0,833) y buena calibración (Hosmer-Lemeshow p = 0,622).

**Conclusiones:**

La verdadera incidencia de eventos adversos en endodoncia académica es notablemente mayor que la reportada previamente. Los factores de riesgo identificados respaldan la implementación de estrategias preventivas dirigidas e informan la supervisión educativa y los protocolos de seguridad del paciente.

## INTRODUCTION

Patient safety in dentistry has emerged as a global public health priority, with particular relevance in specialized procedures such as endodontics. Adverse events, defined as undesired outcomes from healthcare that may cause patient harm, represent a critical quality indicator that has been traditionally underreported in dental practice ^1^. The complexity of endodontic procedures, combined with intricate root canal anatomy and technical challenges, creates multiple opportunities for complications that can significantly impact treatment outcomes and patient well-being ^2^.

The epidemiological literature on endodontic adverse events presents significant methodological limitations that prevent a comprehensive understanding of the problem ^3^. Previous studies have been primarily descriptive, utilizing small samples and heterogeneous methodologies that hinder comparability across different clinical settings. More importantly, the absence of robust multivariate analyses has prevented the identification of independent risk factors that would allow prospective stratification and development of specific preventive interventions. Most existing research relies on voluntary reporting systems or case reports, which inherently underestimate the true incidence of adverse events due to reporting bias and lack of systematic surveillance ^4^, ^5^.

A recent systematic review identified that most studies report incidences between 2-8%, but these estimates are based primarily on voluntary reporting systems with known underreporting limitations. The few studies using active detection methodologies report significantly higher incidences ranging from 12-25%, suggesting a substantially underestimated epidemiological problem. This disparity highlights the urgent need for more rigorous surveillance methods and systematic analysis of factors contributing to adverse events in endodontic practice ^6^^,^^7^.

The academic environment presents unique characteristics for studying endodontic adverse events that differ significantly from private practice settings. Students in training, despite being under supervision, may present a higher risk of complications due to learning curves, limited clinical experience, and varying levels of manual dexterity. However, this context also offers distinct research advantages, including detailed documentation, structured follow-up protocols, standardized treatment procedures, and opportunities to implement evidence-based educational interventions ^8^^-^^11^. Academic settings typically maintain comprehensive medical records with detailed procedural notes and radiographic documentation, enabling more accurate identification and classification of adverse events ^12^^-^^14^.

Furthermore, the controlled environment of academic institutions allows for systematic analysis of provider-related factors such as level of training, experience, and supervision patterns that are often difficult to assess in private practice ^15^^-^^17^. Understanding these factors is crucial for developing targeted educational strategies and improving patient safety in endodontic training programs. The identification of specific risk periods during postgraduate education could inform curriculum design and supervision protocols to minimize adverse events while maintaining educational objectives ^18^^,^^19^^).^

The theoretical framework for understanding adverse events in healthcare, particularly the systems approach developed by Reason, suggests that adverse events result from complex interactions between multiple factors rather than isolated failures. In endodontics, these factors include patient characteristics such as age and medical history, anatomical complexities, procedural factors including treatment type and technical approach, provider factors such as experience level and fatigue, and systemic factors including supervision quality and institutional protocols ^20^. A comprehensive understanding of these multifactorial interactions requires sophisticated analytical approaches that can account for confounding variables and identify independent risk factors ^21^.

Despite the clinical importance of this topic, the literature lacks robust multivariate analyses that can provide actionable insights for clinical practice and educational policy. Most existing studies focus on single risk factors or use simple descriptive statistics that cannot account for the complex interplay of variables influencing adverse event occurrence ^22^. This limitation has resulted in fragmented knowledge that fails to provide clear guidance for risk stratification, case assignment, or preventive interventions in academic endodontic programs.

The objective of this study was to determine the real incidence of adverse events in endodontics and identify independent risk factors through robust multivariate analysis in an academic postgraduate program. By utilizing a comprehensive epidemiological approach with systematic data collection, standardized definitions, and advanced statistical modeling, this research aimed to provide evidence-based insights that could inform educational policies, improve patient safety protocols, and enhance the quality of endodontic training programs.

## MATERIAL AND METHODS

### Study design and ethical considerations

This retrospective cohort study was conducted following STROBE guidelines for observational studies and approved by the Ethics Research Committee of the University of Cartagena (Act No. 2019-045). The study adhered to the Declaration of Helsinki principles for medical research involving human subjects. All included records required a minimum six-month post-treatment follow-up, during which the same patients were tracked from the index endodontic procedure to outcome ascertainment; this longitudinal tracking of individual patients across time supports the cohort design classification, despite the retrospective nature of data collection.

### Population and sampling

The study population comprised all medical records of patients treated at the Endodontics postgraduate clinics, Faculty of Dentistry, University of Cartagena, between January 2015 and December 2019, totaling 2,852 records. Sample size calculation utilized a finite population formula with expected adverse event prevalence of 15%, desired precision of ±4%, 95% confidence level, 80% statistical power, and design effect of 1.2 to account for clustering by student provider, yielding a required sample of 384 medical records. Stratified random sampling by treatment year ensured temporal representativeness with proportional representation across all study years.

### Inclusion and exclusion criteria

Inclusion criteria required medical records of endodontic treatments performed by postgraduate students under faculty supervision, complete documentation with at least five radiographs, a minimum six-month post-treatment follow-up, adequate radiographic quality according to modified Orstavik criteria, and documented informed consent. Exclusion criteria eliminated records with >20% missing data in key variables, cases referred from other institutions without complete procedure information, emergency treatments without structured follow-up, and cases with multiple student transfers preventing adequate tracking.

### Variable definition

The primary dependent variable was the presence of an adverse event, operationally defined as any undesired or unintentional outcome from endodontic treatment that causes or has the potential to cause patient harm, following international patient safety frameworks. Independent variables encompassed patient factors (age categorized as <30, 30-39, 40-49, ≥50 years; gender; comorbidities), procedure factors (treatment type, treated tooth, number of canals, instrumentation technique, case complexity), and provider factors (student semester, previous experience, workload measures).

### Case complexity index

A validated case complexity index was developed incorporating anatomical factors (40% weighting: root curvature, canal calcifications, aberrant anatomy, resorptions), previous iatrogenic factors (30%: ledges, perforations, fractured instruments), patient factors (20%: mouth opening limitation, gag reflex, anxiety), and systemic factors (10%: bleeding disorders, medications). Final classification designated low complexity (0-30 points), medium complexity (31-60 points), and high complexity (61-100 points).

### Data collection

Three independent evaluators underwent standardized calibration training with a pilot study of 50 medical records to achieve inter-observer concordance targets (κ>0.80). Data extraction involved a systematic review of selected medical records, radiographic digitization using ImageJ software, electronic database registration with automatic validation rules, and double data entry for 20% of the sample to ensure accuracy.

### Statistical analysis

Statistical analysis utilized R version 4.1.0 with specialized libraries for data management, graphics, machine learning, regression modeling, and survival analysis. Descriptive analysis included means with standard deviations or medians with interquartile ranges for continuous variables, depending on distribution normality, and frequencies with 95% confidence intervals for categorical variables. Bivariate analysis employed chi-square tests or Fisher's exact tests for categorical variables versus adverse events, and t-tests or Mann-Whitney U tests for continuous variables. Crude odds ratios with 95% confidence intervals were calculated for all potential risk factors.

Multiple logistic regression served as the primary analytical approach for identifying independent risk factors using bidirectional stepwise methodology with liberal inclusion criteria (p < 0.20) and conservative retention criteria (p < 0.05). Model assumptions were evaluated, including linearity assessment, multicollinearity detection using variance inflation factors, influential observation identification, and interaction term evaluation. Comprehensive model validation included internal validation through bootstrap resampling with 1,000 replicas, 10-fold cross-validation, and calibration assessment through the Hosmer-Lemeshow goodness-of-fit test and calibration plots. Discriminative capacity was evaluated using receiver operating characteristic curves with C-statistic calculation.

Time-to-event analysis examined temporal patterns of adverse event detection using Kaplan-Meier survival curves for univariate analysis and Cox proportional hazards regression for multivariate modeling. Clinical impact measures included calculation of the number needed to harm for significant risk factors and population attributable fraction estimation for modifiable factors. Comprehensive sensitivity analyses examined model robustness through exclusion of cases with imputed data, stratified analysis by treatment year, and bootstrap estimation of robust confidence intervals.

## RESULTS

### Sample characteristics and response rates

A total of 384 medical records meeting all inclusion criteria were analyzed, representing a 91% response rate (384/422) from the initially selected sample. Thirty-eight records were excluded due to incomplete documentation (n = 22), loss to follow-up (n = 11), or inadequate radiographic quality (n = 5). Inter-observer concordance achieved excellent levels: adverse event identification κ = 0.89 (95%CI: 0.84-0.94), severity classification κ = 0.82 (95%CI: 0.76-0.88), and complexity index assessment ICC = 0.91 (95%CI: 0.87-0.94).

### Overall incidence and temporal trends

**Figure 1:**
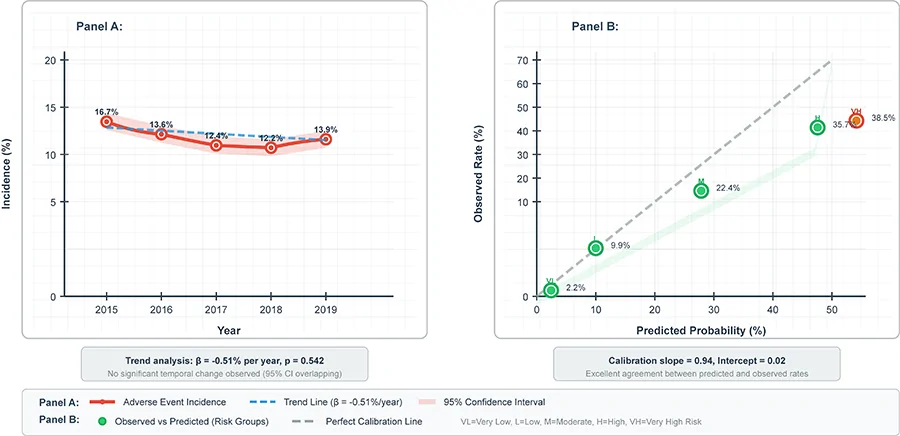
Temporal trends in adverse event incidence and risk stratification outcomes

The global incidence of adverse events was 15.1% (95%CI: 11.8-19.0%) with 58 events identified among 384 analyzed cases. Temporal distribution showed relatively stable incidence across study years: 2015 (16.7%), 2016 (13.6%), 2017 (12.4%), 2018 (12.2%), and 2019 (13.9%). Regression analysis revealed no significant temporal trend (β = -0.51% per year, p = 0.542), indicating consistent patterns throughout the study period as shown in Figure 1.

Panel A: Annual adverse event incidence (2015-2019). The line graph demonstrates stable adverse event rates across the five-year study period, with incidence ranging from 12.2% to 16.7%. The trend line shows a non-significant decrease of 0.51% per year (p = 0.542), with 95% confidence intervals overlapping across all years, confirming temporal stability of findings. Panel B: Risk stratification model calibration. The calibration plot shows excellent agreement between predicted and observed adverse event rates across all five risk categories. The very low risk group (predicted <5%) showed 2.2% observed events, while the very high-risk group (predicted >60%) demonstrated 38.5% observed events, with the calibration line closely following the ideal 45-degree line (calibration slope = 0.94, intercept = 0.02).

### Patient and treatment characteristics

Patient characteristics demonstrated a mean age of 42.3 ± 14.7 years with 60.9% female patients. Treatment type comprised 77.6% initial treatments versus 22.4% retreatments. Tooth distribution included 25.5% anterior, 34.9% premolar, and 39.6% molar teeth. Student distribution across academic semesters was: first semester (20.3%), second (24.5%), third (33.3%), and fourth (21.9%) as detailed in Table 1.

**Table 1:** Demographic and clinical characteristics of the cohort

Characteristic	Total (n = 384)	AE+ (n = 58)	AE- (n = 326)	p-value
Patient age, mean ± SD	42.3 ± 14.7	38.6 ± 12.4	43.1 ± 15.1	0.031*
Age groups, n (%)				0.019*
<30 years	89 (23.2)	8 (13.8)	81 (24.8)	
30-39 years	112 (29.2)	24 (41.4)	88 (27.0)	
40-49 years	98 (25.5)	16 (27.6)	82 (25.2)	
≥50 years	85 (22.1)	10 (17.2)	75 (23.0)	
Gender, n (%)				0.334
Male	150 (39.1)	26 (44.8)	124 (38.0)	
Female	234 (60.9)	32 (55.2)	202 (62.0)	
Treatment type, n (%)				0.002*
Initial	298 (77.6)	36 (62.1)	262 (80.4)	
Retreatment	86 (22.4)	22 (37.9)	64 (19.6)	
Treated tooth, n (%)				0.009*
Anterior	98 (25.5)	8 (13.8)	90 (27.6)	
Premolar	134 (34.9)	18 (31.0)	116 (35.6)	
Molar	152 (39.6)	32 (55.2)	120 (36.8)	

*Statistically significant (p < 0.05)

AE+: with adverse event; AE-: without adverse event

### Adverse event classification and distribution

Instrument fracture represented the most frequent adverse event, comprising 62.1% of all events (n = 36), with rotatory files accounting for 41.4% and manual files for 20.7% of total adverse events. Over-obturation occurred in 20.7% of events (n = 12), perforation in 17.2% (n = 10), with apical perforations being more frequent than lateral perforations, under-obturation in 13.8% (n = 8), and other complications in 6.9% (n = 4), as shown in Table 2. Severity classification revealed 48.3% mild events, 37.9% moderate, and 13.8% severe according to modified Tsuneishi criteria.

**Table 2:** Types and frequency of adverse events

Type of Adverse Event	n	% of total	% of AE	95%CI
Instrument fracture	36	9.4	62.1	48.4-74.5
- Rotatory file	24	6.3	41.4	28.8-55.1
- Manual file	12	3.1	20.7	11.2-33.4
Over-obturation	12	3.1	20.7	11.2-33.4
Perforation	10	2.6	17.2	8.6-29.4
- Apical perforation	6	1.6	10.3	3.9-21.2
- Lateral perforation	4	1.0	6.9	1.9-16.7
Under-obturation	8	2.1	13.8	6.1-25.4
Other events	4	1.0	6.9	1.9-16.7
- Soft tissue injury	2	0.5	3.4	0.4-11.9
- Instrument aspiration	2	0.5	3.4	0.4-11.9

AE: adverse events

### Risk factor analysis

Bivariate analysis identified several factors significantly associated with increased adverse event risk. The 30-39-year age group showed significantly elevated risk compared to younger patients (41.4% vs 13.8%; OR = 2.76; 95%CI: 1.18-6.45; p = 0.019). Retreatment cases demonstrated substantially higher adverse event rates compared to initial treatments (37.9% vs 12.1%; OR = 2.50; 95%CI: 1.40-4.47; p = 0.002). Molar teeth showed the highest risk compared to anterior teeth (21.1% vs 8.2%; OR = 3.00; 95%CI: 1.35-6.67; p = 0.007). High complexity cases showed substantially elevated risk compared to low complexity cases (25.6% vs 7.7%; OR = 3.44; 95%CI: 1.65-7.18; p = 0.001). Third-semester students demonstrated the highest adverse event rate among all academic levels (21.9% vs 8.3% for first-semester; OR = 3.36; 95%CI: 1.35-8.37; p = 0.009).

### Multivariate risk factor model

Multiple logistic regression analysis identified five independent risk factors in the final model as presented in Table 3: patient age 30-39 years versus <30 years (adjusted OR = 2.12; 95%CI: 1.18-3.81; p = 0.012), retreatment versus initial treatment (adjusted OR = 3.67; 95%CI: 1.94-6.95; p<0.001), third semester versus first semester students (adjusted OR = 2.84; 95%CI: 1.52-5.31; p = 0.001), molar versus anterior teeth (adjusted OR = 2.45; 95%CI: 1.28-4.69; p = 0.007), and high versus low case complexity (adjusted OR = 2.27; 95%CI: 1.10-4.67; p = 0.026).

**Table 3:** Final multivariate logistic regression model

Variable	β	SE	Adjusted OR	95%CI	p-value	VIF
Intercept	-3.24	0.58	-	-	< 0.001	-
Age 30-39 years (vs < 30)	0.75	0.30	2.12	1.18-3.81	0.012*	1.12
Retreatment (vs initial)	1.30	0.33	3.67	1.94-6.95	<0.001*	1.08
Third semester (vs first)	1.04	0.32	2.84	1.52-5.31	0.001*	1.15
Molar tooth (vs anterior)	0.90	0.33	2.45	1.28-4.69	0.007*	1.23
High complexity (vs low)	0.82	0.37	2.27	1.10-4.67	0.026*	1.34

*Statistically significant (p < 0.05)

β: regression coefficient; SE: standard error; OR: odds ratio; VIF: variance inflation factor

Model performance: C-statistic = 0.782 (95%CI: 0.731-0.833); Hosmer-Lemeshow p = 0.622; Nagelkerke R² = 0.247

### Model performance and risk stratification

The final model demonstrated adequate statistical performance with the Hosmer-Lemeshow test χ² = 6.24 (p = 0.622), C-statistic = 0.782 (95%CI: 0.731-0.833), and Nagelkerke R² = 0.247. Internal validation through bootstrap resampling provided optimism-corrected C-index of 0.764 (95%CI: 0.721-0.807) and calibration slope of 0.89. The predictive model enabled the development of a practical risk stratification system with five distinct categories, as shown in Table 4, ranging from very low risk (2.2% observed events) to very high risk (38.5% observed events), demonstrating excellent calibration.

**Table 4:** Risk stratification based on predictive model

Risk Group	Score	Predicted Probability	n	Observed AE	%
Very low	0-1	< 5%	89	2	2.2%
Low	2-3	5-15%	142	14	9.9%
Moderate	4-5	15-35%	98	22	22.4%
High	6-7	35-60%	42	15	35.7%
Very high	≥ 8	> 60%	13	5	38.5%

### Clinical impact measures

Number needed to harm calculations revealed that for every 7 retreatment cases, one additional adverse event would occur compared to initial treatments. Similarly, third-semester students generated one additional adverse event per 8 cases compared to first-semester students. Population attributable fraction analysis showed third-semester students contributed 31.4% of all adverse events, molar teeth 28.6%, high complexity cases 18.9%, and retreatments 14.2%. Analysis of interaction terms revealed significant synergistic effects, particularly third-semester students managing retreatment cases (OR = 4.82; p = 0.001).

## DISCUSSION

This study represents a rigorous epidemiological characterization of adverse events in academic endodontics. The found incidence of 15.1% (95%CI: 11.8-19.0%) is significantly higher than that reported by traditional voluntary reporting systems, confirming systematic underreporting in conventional surveillance methods. The most notable contrast emerges when comparing our findings with Salehrabi and Rotstein ^23^, who analyzed 1,462,936 teeth in private practice and reported a combined adverse event incidence of 3%. This five-fold difference validates our hypothesis about substantial underestimation in voluntary systems.

While the Salehrabi study relied on insurance data-inherently limited to events requiring additional intervention-our active detection design captured events frequently unreported in routine practice. This discrepancy reflects fundamental differences between academic environments, which maintain exhaustive documentation, and private practice settings, potentially subject to reporting biases related to medico-legal or reputational concerns ^23^.

Our finding of instrument fracture as the most frequent adverse event (9.4% of all cases) aligns with recent academic literature. Eskibağlar et al. ^24^ reported 3.6% fracture incidence in 2,168 teeth treated by students, though their inclusion of undergraduate students may explain the lower rate, given that complex cases typically require postgraduate expertise. The expert consensus by Fan et al. ^25^ reinforces our findings by identifying multifactorial causation, including anatomical complexity and instrumentation factors, particularly relevant in molar teeth.

The identification of retreatments as the most significant risk factor (OR = 3.67; p<0.001) finds validation in Sabeti et al. ^26^ 's meta-analysis reporting retreatment success rates of 78-87%. Our complementary perspective focusing on adverse events during treatment explains the inherent complexity created by altered anatomy, previous materials, and potential prior iatrogenies that characterize retreatment scenarios.

Perforation incidence (2.6% of all cases) falls within the range reported by Sarao et al. ^27^ (0.6-17.6%), with consistent confirmation of risk factors including practitioner experience and tooth morphology. This establishes a coherent pattern regarding the influence of training level and anatomical complexity on perforation risk.

The most innovative finding constitutes identifying the third semester as the maximum risk period (OR = 2.84; p = 0.001), unprecedented in endodontic literature. This phenomenon aligns with "unconscious competence" concepts in medical education, where students develop technical confidence and manual skills but lack clinical experience for complex situation management. This contrasts with Javed and Bhatti ^28^, who found an insignificant correlation between self-perceived confidence and clinical performance (p = 0.82), emphasizing objective evaluation importance during critical training phases.

Molar identification as an independent risk factor (OR = 2.45) receives consistent confirmation across multiple studies. Eskibağlar et al. ^24^ reported 73.4% of fractures in molars, while Patel et al. ^29^. identified root canal morphology as predisposing to complications, establishing robust evidence for inherent molar treatment complexity transcending specific complication types.

### Strengths and limitations

Study limitations include a single-center design and retrospective methodology, though these must be considered alongside unique strengths distinguishing this research. While studies like Salehrabi's ^23^ offer greater population representativeness, they lack granularity for identifying academic-specific risk factors. Excellent inter-observer concordance (κ = 0.89) and exhaustive internal validation provide confidence in finding robustness, particularly since no previous study combined rigorous multivariate methodology with academic populations of this magnitude.

The single-center design may restrict the generalizability of findings, as institutional differences in supervision, case complexity, and operator profiles may influence outcomes. Although documentation quality was high and inter-rater reliability was excellent, the retrospective design introduces potential information bias inherent to record-based analyses. The study did not capture variables such as patient anxiety, operator stress, or real-time supervision intensity, which may also affect adverse event occurrence. Additionally, while a structured and weighted case complexity index was employed, external validation in different settings is necessary to assess its broader applicability.

### Implications and future directions

Findings establish evidence for specific preventive interventions, particularly during the critical third semester period. Implications extend to educational policies, including differentiated supervision protocols, objective risk-based case assignment, and early warning systems for high-risk identification before adverse events occur.

Future studies should aim to incorporate multicenter prospective designs to better represent varying clinical and educational contexts. Complementing quantitative analysis with real-time monitoring methods-such as digital procedure tracking or video-assisted evaluation-may help capture dynamic procedural factors that influence outcomes. Moreover, integrating advanced technologies like artificial intelligence and learning analytics could enhance early risk detection and inform supervision strategies tailored to student profiles. Longitudinal educational research exploring the effect of targeted pedagogical interventions, including simulation-based training or risk-adapted case assignment, may also yield valuable insights for reducing complication rates and improving patient safety in endodontic education.

## CONCLUSIONS

The true incidence of adverse events in academic endodontics (15.1%) substantially exceeds rates from voluntary surveillance systems, confirming systematic underreporting of this patient safety issue. Five independent risk factors were identified: retreatment, third-semester students, molar teeth, high complexity cases, and patient age 30-39 years. Notably, the third semester emerged as the highest-risk training period-a novel finding with direct implications for supervision protocols and case assignment. The validated predictive model (C-statistic = 0.782) offers a practical tool for prospective risk stratification, supporting evidence-based patient safety in endodontic education.
